# Association Between Early Recovery of Kidney Function After Acute Kidney Injury and Long-term Clinical Outcomes

**DOI:** 10.1001/jamanetworkopen.2020.2682

**Published:** 2020-04-13

**Authors:** Pavan K. Bhatraju, Leila R. Zelnick, Vernon M. Chinchilli, Dennis G. Moledina, Steve G. Coca, Chirag R. Parikh, Amit X. Garg, Chi-yuan Hsu, Alan S. Go, Kathleen D. Liu, T. Alp Ikizler, Edward D. Siew, James S. Kaufman, Paul L. Kimmel, Jonathan Himmelfarb, Mark M. Wurfel

**Affiliations:** 1 Division of Pulmonary, Critical Care and Sleep Medicine, Department of Medicine, University of Washington, Seattle; 2 Kidney Research Institute, Division of Nephrology, Department of Medicine, University of Washington, Seattle; 3 Penn State College of Medicine, Department of Public Health Sciences, Hershey, Pennsylvania; 4 Section of Nephrology, Department of Internal Medicine, Yale School of Medicine, New Haven, Connecticut; 5 Program of Applied Translational Research, Department of Internal Medicine, Yale School of Medicine, New Haven, Connecticut; 6 Section of Nephrology, Department of Internal Medicine, Mount Sinai School of Medicine, New York, New York; 7 Division of Nephrology, School of Medicine, Johns Hopkins University, Baltimore, Maryland; 8 Division of Nephrology, Department of Medicine, Western University, London, Ontario, Canada; 9 Division of Nephrology, Department of Medicine, University of California, San Francisco; 10 Division of Research, Kaiser Permanente Northern California, Oakland; 11 Department of Epidemiology and Biostatistics, University of California, San Francisco; 12 Division of Critical Care, Department of Anesthesia, University of California, San Francisco; 13 Division of Nephrology and Hypertension, Vanderbilt University Medical Center, Nashville, Tennessee; 14 Division of Nephrology, New York University School of Medicine, New York; 15 Division of Nephrology, Veterans Affairs New York Harbor Healthcare System, New York; 16 Division of Renal Diseases and Hypertension, Department of Medicine, George Washington University Medical Center, Washington, DC

## Abstract

**Importance:**

The severity of acute kidney injury (AKI) is usually determined based on the maximum serum creatinine concentration. However, the trajectory of kidney function recovery could be an additional important dimension of AKI severity.

**Objective:**

To assess whether the trajectory of kidney function recovery within 72 hours after AKI is associated with long-term risk of clinical outcomes.

**Design, Setting, and Participants:**

This prospective, multicenter cohort study enrolled 1538 adults with or without AKI 3 months after hospital discharge between December 1, 2009, and February 28, 2015. Statistical analyses were completed November 1, 2018. Participants with or without AKI were matched based on demographic characteristics, site, comorbidities, and prehospitalization estimated glomerular filtration rate. Participants with AKI were classified as having resolving or nonresolving AKI based on previously published definitions. Resolving AKI was defined as a decrease in serum creatinine concentration of 0.3 mg/dL or more or 25% or more from maximum in the first 72 hours after AKI diagnosis. Nonresolving AKI was defined as AKI not meeting the definition for resolving AKI.

**Main Outcomes and Measures:**

The primary outcome was a composite of major adverse kidney events (MAKE), defined as incident or progressive chronic kidney disease, long-term dialysis, or all-cause death during study follow-up.

**Results:**

Among 1538 participants (964 men; mean [SD] age, 64.6 [12.7] years), 769 (50%) had no AKI, 475 (31%) had a resolving AKI pattern, and 294 (19%) had a nonresolving AKI pattern. After a median follow-up of 4.7 years, the outcome of MAKE occurred in 550 (36%) of all participants. The adjusted hazard ratio for MAKE was higher for patients with resolving AKI (adjusted hazard ratio, 1.52; 95% CI, 1.01-2.29; *P* = .04) and those with nonresolving AKI (adjusted hazard ratio 2.30; 95% CI, 1.52-3.48; *P* < .001) compared with participants without AKI. Within the population of patients with AKI, nonresolving AKI was associated with a 51% greater risk of MAKE (95% CI, 22%-88%; *P* < .001) compared with resolving AKI. The higher risk of MAKE among patients with nonresolving AKI was explained by a higher risk of incident and progressive chronic kidney disease.

**Conclusions and Relevance:**

This study suggests that the 72-hour period immediately after AKI distinguishes the risk of clinically important kidney-specific long-term outcomes. The identification of different AKI recovery patterns may improve patient risk stratification, facilitate prognostic enrichment in clinical trials, and enable recognition of patients who may benefit from nephrology consultation.

## Key Points

**Question:**

Is the trajectory of kidney function within 72 hours after acute kidney injury associated with 5-year clinical outcomes, such as chronic kidney disease, dialysis, and death?

**Findings:**

Among 1538 participants in this prospective multicenter cohort study, the early recovery pattern after acute kidney injury was associated with long-term outcomes. In adjusted analyses, patients with a nonresolving recovery pattern after acute kidney injury had a 51% greater risk for the composite kidney-specific clinical outcome compared with patients with a resolving acute kidney injury recovery pattern, independent of traditional criteria to risk stratify patients with acute kidney injury.

**Meaning:**

This study’s finding suggest that the acute recovery pattern after development of acute kidney injury should be considered in evaluating the risk of long-term clinical outcomes.

## Teaser

This cohort study assesses whether the trajectory of kidney function recovery within 72 hours after acute kidney injury is associated with long-term risk of clinical outcomes.

## Introduction

Acute kidney injury (AKI) is common in the hospital setting,^1^ costs $10 billion annually in the United States,^2^ and is associated with poor long-term outcomes.^3,4,5^ Acute kidney injury is defined by the Kidney Disease: Improving Global Outcomes (KDIGO) consensus group as an increase in the concentration of serum creatinine (SCr) of 0.3 mg/dL or more (to convert to micromoles per liter, multiply by 88.4) or 50% or more of the baseline within a 48-hour period or within 7 days after hospitalization or a decrease in urine output.^6^ The KDIGO group classifies severity of AKI from stage 0 (no AKI) to stage 3 based on the maximum change in SCr concentration or the minimum urine output throughout the hospital stay. However, the KDIGO definition does not stratify patients based on differences in AKI recovery patterns. Combining patients with different AKI recovery patterns may hide subgroups that are more tightly associated with clinical outcomes^7^ and may conceal unique pathophysiological processes specific to certain populations with AKI.^8,9,10,11^

In AKI, it has been particularly problematic to identify reproducible subgroups with distinct clinical outcomes. For example, clinicians have historically separated AKI into prerenal AKI and acute tubular necrosis.^12,13^ However, this distinction has several limitations. First, urine microscopy to diagnose acute tubular necrosis can be dependent on the timing of urine sample collection during the course of AKI. Second, the sensitivity of urine microscopy may vary based on AKI risk factors.^14,15^ Third, the fractional excretion of sodium has poor reliability to differentiate between these 2 groups.^16,17^ Fourth, even with clinical adjudication by expert nephrologists, there is poor agreement between diagnoses of acute tubular necrosis vs prerenal AKI.^18^ Thus, we and others have sought to identify subgroups of patients with AKI based on kidney functional recovery after injury.^19,20,21^

The trajectory of renal dysfunction is a potentially important and clinically intuitive parameter by which to risk stratify AKI. When stratifying AKI based on trajectory, a patient’s response to early medical interventions is considered, and this information provided by serial measures of renal dysfunction is used to identify AKI recovery subgroups. In addition, the trajectory is an assessment, early after AKI diagnosis, that could be associated with therapeutic decisions in the hospital or early after hospital discharge.

Although AKI recovery patterns stratify the risk of poor short-term outcomes,^19,20^ to date, it is unknown whether these same AKI recovery subgroups differentiate the risk for long-term kidney-specific clinical outcomes. Our primary outcome was a composite of major adverse kidney events (MAKE), which included the development or progression of chronic kidney disease (CKD), the initiation of long-term dialysis, or death from any cause,^22^ during follow-up. We hypothesized a graded association, with nonresolving AKI having the highest risk of MAKE, then resolving AKI, and finally participants without AKI having the least risk for MAKE. We examined this hypothesis in the Assessment, Serial Evaluation, and Subsequent Sequelae (ASSESS-AKI) cohort, a large, multiethnic observational cohort with a median follow-up of approximately 4.7 years.

## Methods

### Study Population

The ASSESS-AKI Study, sponsored by the National Institute of Diabetes and Digestive and Kidney Diseases, is a prospective cohort study of hospitalized persons who did or did not experience an episode of AKI and survived to complete a baseline study visit 3 months after hospital discharge. Detailed eligibility criteria are included in the eMethods in the Supplement and have been previously published.^23^ Acute kidney injury during the index hospitalization was defined using modified KDIGO criteria^6^ based on an increase in SCr concentration of 50% or more or 0.3 mg/dL or more above an outpatient, non–emergency department baseline value within 7 to 365 days before the index admission. Study participants were then prospectively followed up and included if they survived at least 3 months after the index hospitalization. The present study was a preplanned study of ASSESS-AKI. This study followed the Strengthening the Reporting of Observational Studies in Epidemiology (STROBE) reporting guideline. The study was approved by the Yale University, Vanderbilt University, Kaiser Permanente, and University of Washington Institutional Review Boards. Written informed consent was obtained from participants.

Resolving AKI was defined as a decrease in the concentration of SCr of 0.3 mg/dL or more or 25% or more from maximum in the first 72 hours after AKI diagnosis. Nonresolving AKI was defined as all AKI cases not meeting the definition of resolving AKI.^19,20^ If participants were discharged from hospital before 72 hours after AKI diagnosis, then the last SCr measurement prior to hospital discharge was used to determine criteria for resolving or nonresolving AKI. All participants with resolving AKI had to have a sustained decrease in SCr concentration during the 72-hour time window.

In ASSESS-AKI, study participants with or without AKI were matched based on demographic characteristics, hospital factors, and baseline kidney function. Further details can be found in the eMethods in the Supplement. Matching was not completed among patients without AKI compared with those with resolving or nonresolving AKI. Thus, patient characteristics may be unbalanced between the resolving and nonresolving AKI groups.

### Follow-up Study Visits and Ascertainment of Outcomes

Follow-up study visits were conducted 3 and 12 months after the index hospitalization and annually thereafter (with determination of estimated glomerular filtration rate [eGFR]), with interim telephone contacts at approximately 6-month intervals.^24^ Medical history, including interim hospitalizations, and medication use were updated at each contact. Follow-up time was defined as the difference between the event or censoring date and the 3-month visit, with outcomes censored owing to withdrawal, loss to follow-up, or end of the study. Outcomes were ascertained through September 1, 2018. Vital status was updated at each study contact and through medical records review.

Our primary outcome was MAKE during a median study follow-up of 4.7 years starting at the time of baseline study visit at 3 months after hospital discharge.^22,24^ We chose MAKE to account for issues of competing risk and to use an outcome that considers the breadth of clinical outcomes after AKI. Incident CKD among participants without preexisting CKD prior to the index hospitalization was defined as a 25% or greater reduction in eGFR compared with the 3-month posthospitalization measured eGFR and achieving CKD stage 3 or higher.^24^ Progression of CKD in participants with preexisting CKD at the index hospitalization (preadmission eGFR, <60 mL/min/1.73 m^2^) was defined as a 50% or greater reduction in eGFR compared with the 3-month posthospitalization eGFR, reaching CKD stage 5 or receiving renal replacement therapy (long-term dialysis or kidney transplant).

### Covariates

Demographic characteristics included age, sex, and self-reported race/ethnicity (white, black, or other) and Hispanic race/ethnicity. We recorded self-reported prior cardiovascular disease (heart failure, myocardial infarction, stroke, or peripheral artery disease). Hypertension was based on self-report combined with taking antihypertensive agents, or having a study visit systolic blood pressure greater than 140 mm Hg and/or a diastolic blood pressure greater than 90 mm Hg. Diabetes (types 1 and 2) was based on self-report, receipt of antidiabetic agents, or having a glycosylated hemoglobin level of 6.5% or greater (to convert to proportion of total hemoglobin, multiply by 0.01). Sepsis was based on suspected infection plus the presence of at least 2 criteria of systemic inflammatory response syndrome. Shock was defined by physician diagnosis. A serum creatinine concentration returning to baseline was defined as the SCr measurement at hospital discharge or at 3 months being equal to or lower than the prehospitalization SCr measurement. There were no missing data in any of the primary exposures of interest, other key covariates, or outcomes examined.

### Statistical Analysis

Statistical analyses were completed November 1, 2018. We summarized baseline participant characteristics across groups with no AKI, resolving AKI, and nonresolving AKI, with mean (SD) values for continuous variables and number and percentage for categorical variables. For the primary analysis, we used Cox proportional hazards regression together with infinitesimal jackknife SEs to account for correlation within matched AKI and no AKI pairs.^25^ We evaluated the association of AKI subgroups (resolving and nonresolving) with incident MAKE during a median of 4.7 years (interquartile range, 3.4-5.7 years), and follow-up was censored at the end of administrative follow-up, loss to follow-up, or death. Because matching was not completed between patients with resolving AKI and patients with nonresolving AKI, we completed a series of a priori nested models controlling for potential confounding factors: age, sex, black race, diabetes, CKD status, cardiovascular disease, sepsis, center enrollment site, mechanical ventilation, diagnosed shock, major surgery, and KDIGO stage of AKI at 72 hours after AKI diagnosis. We tested the Cox proportional hazards regression assumption and found a statistically significant violation. In response, we inspected the Schoenfield residuals and chose a cutoff of 3 years on the basis of when risk seemed to differ across time; in a sensitivity analysis, we repeated the primary analysis, allowing for differing associations during years 0 to 3 and years 3 through the end of follow-up. For all analyses, a 2-tailed *P* < .05 was taken as evidence of statistical significance. All statistical analyses were performed in R, version 3.6.0 (R Project for Statistical Computing).

## Results

### Participant Characteristics

Of 1538 hospitalized participants (964 men; mean [SD] age, 64.6 [12.7] years) in ASSESS-AKI, 769 (50%) had no AKI, 475 (31%) had a resolving AKI pattern, and 294 (19%) had a nonresolving AKI pattern (Table 1). The mean (SD) maximum concentration of SCr in the first 72 hours after AKI diagnosis was 1.1 (0.4) mg/dL in the no AKI population, 2.4 (1.5) mg/dL in the resolving AKI group, and 2.4 (1.8) mg/dL in the nonresolving AKI group. The differences in SCr concentration between patients with resolving AKI and patiens with nonresolving AKI at baseline and within 72 hours after AKI diagnosis were not significant. Participants with nonresolving AKI were more likely than those with resolving AKI to be men (206 [70%] vs 313 [66%]), have diabetes (156 [53%] vs 231 [49%]), and have preexisting CKD (120 [41%] vs 186 [39%]). In contrast, participants with resolving AKI were more likely than those with nonresolving AKI to have sepsis (86 [18%] vs 32 [11%]) and KDIGO stage 2 AKI (87 [18%] vs 36 [12%]) or stage 3 AKI (52 [11%] vs 28 [10%]). Of the 769 patients with AKI, 566 (74%) had KDIGO stage 1 AKI.

**Table 1.** Baseline Characteristics by AKI Recovery Patterns

Variable	Overall (N = 1538)	No AKI (n = 769)	Resolving AKI (n = 475)	Nonresolving AKI (n = 294)	*P* value^a^
Patients at center, No. (%)					
Yale	308 (20)	154 (20)	75 (16)	79 (27)	.001
Vanderbilt	502 (33)	251 (33)	151 (32)	100 (34)	
Kaiser Permanente	312 (20)	156 (20)	96 (20)	60 (20)	
University of Washington	416 (27)	208 (27)	153 (32)	55 (19)	
Age, mean (SD), y	64.6 (12.7)	65.4 (12.6)	63.2 (12.7)	64.5 (12.8)	.15
Male sex, No. (%)	964 (63)	445 (58)	313 (66)	206 (70)	.24
Black race, No. (%)	204 (13)	81 (11)	79 (17)	44 (15)	.61
BMI, mean (SD)	31.0 (7.7)	30.5 (7.0)	31.2 (8.1)	32.1 (8.7)	.16
Types 1 and 2 diabetes, No. (%)	658 (43)	271 (35)	231 (49)	156 (53)	.24
Chronic kidney disease, No. (%)	612 (40)	306 (40)	186 (39)	120 (41)	.65
History of proteinuria, No. (%)	111 (7)	35 (5)	42 (9)	34 (12)	.40
Congestive heart failure, No. (%)	327 (21)	122 (16)	120 (25)	85 (29)	.28
Sepsis, No. (%)	144 (9)	26 (3)	86 (18)	32 (11)	.007
Use of vasopressors, No. (%)	485 (32)	215 (28)	142 (30)	128 (44)	<.001
Intravenous contrast administered, No. (%)	349 (23)	183 (24)	110 (23)	56 (19)	.21
Major surgical procedure, No. (%)	698 (45)	385 (50)	165 (35)	148 (50)	<.001
Shock, No. (%)	114 (7)	26 (3)	66 (14)	22 (7)	.007
Acute heart failure, No. (%)	76 (5)	17 (2)	34 (7)	25 (9)	.49
Mechanical ventilation, No. (%)	66 (4)	12 (2)	18 (4)	36 (12)	<.001
Acute myocardial infarction, No. (%)	51 (3)	21 (3)	20 (4)	10 (3)	.70
Baseline outpatient serum creatinine concentration, mean (SD), mg/dL	1.2 (0.5)	1.1 (0.4)	1.2 (0.5)	1.3 (0.6)	.37
Baseline outpatient eGFR, mean (SD), mL/min/1.73 m^2^	68.7 (25.0)	70.2 (24.1)	67.8 (25.7)	66.2 (26.0)	.42
Maximum serum creatinine concentration within 72 h of AKI diagnosis, mean (SD), mg/dL	1.7 (1.3)	1.1 (0.4)	2.4 (1.5)	2.4 (1.8)	.86
Serum creatinine concentration at hospital discharge, mean (SD), mg/dL	1.2 (0.8)	1.0 (0.4)	1.3 (0.7)	1.7 (1.2)	<.001
Maximum KDIGO stage of AKI within 72 h after AKI diagnosis, No. (%)					
0	769 (50)	769 (100)	0	0	.05
1	566 (37)	0	336 (71)	230 (78)	NA
2	123 (8)	0	87 (18)	36 (12)	NA
3	80 (5)	0	52 (11)	28 (10)	NA
Length of hospital stay, median (IQR), d	5 (3-8)	4 (3-7)	6 (3-8)	8 (5-13)	<.001

Abbreviations: AKI, acute kidney injury; BMI, body mass index (calculated as weight in kilograms divided by height in meters squared); eGFR, estimated glomerular filtration rate; IQR, interquartile range; KDIGO, Kidney Disease: Improving Global Outcomes; NA, not applicable.

SI conversion factor: To convert to creatinine to micromoles per liter, multiply by 88.4.

^a^ Comparing the outcome of AKI recovery between patients with resolving AKI and patients with nonresolving AKI.

### Renal Recovery by AKI Recovery Patterns

Of the 475 participants with resolving AKI, only 257 (54%) had an SCr concentration that returned to prehospitalization baseline (ie, full AKI recovery) at the time of hospital discharge (Table 2). By 3 months after hospitalization, slightly fewer patients (242 [51%]) had recovered to baseline kidney function in the resolving AKI group. In contrast, a smaller percentage of the 294 participants with a nonresolving AKI had an SCr concentration that returned to baseline at hospital discharge or 3 months after hospitalization. For example, of the participants with nonresolving AKI, only 46 (16%) had AKI recovery by hospital discharge, and 111 (38%) had AKI recovery by 3 months after hospitalization.

**Table 2.** Serum Creatinine Concentration at Hospital Discharge and 3 Months After Hospitalization Stratified by Resolving and Nonresolving AKI

AKI Recovery Pattern	Patients with serum creatinine concentration back to baseline at hospital discharge, No. (%)	*P* value^a^	Patients with serum creatinine concentration back to baseline 3 mo after hospitalization, No. (%)	*P* value^a^
Resolving AKI (n = 475)	257 (54)	<.001	242 (51)	<.001
Nonresolving AKI (n = 294)	46 (16)		111 (38)	

Abbreviation: AKI, acute kidney injury.

^a^ Comparing the outcome of AKI recovery between patients with resolving AKI and patients with nonresolving AKI.

### Associations of AKI Recovery Patterns With MAKE

The primary outcome of MAKE occurred in 550 (36%) of all participants in ASSESS-AKI. The unadjusted incidence rate for MAKE was 5.9 events per 100 patient-years among participants without AKI, 11.9 events per 100 patient-years among those with resolving AKI, and 16.6 events per 100 patient-years among those with nonresolving AKI. Kaplan-Meier estimates of the proportion of participants experiencing kidney-related events by AKI recovery subgroups at 4 years of follow-up is provided in Table 3. After adjustment for baseline demographic characteristics, diabetes, cardiovascular disease, CKD, sepsis, and site of enrollment, the adjusted hazard ratio (aHR) for MAKE was higher in both the resolving (aHR, 1.95; 95% CI, 1.58-2.40; *P* < .001) and nonresolving (aHR, 2.80; 95% CI, 2.26-3.46; *P* < .001) AKI groups compared with hospitalized participants without AKI (Table 4). Additional adjustment for KDIGO stage of AKI at 72 hours after AKI diagnosis, shock, mechanical ventilation, and major surgery showed these associations to persist, with a higher risk of MAKE in both the resolving (aHR, 1.52; 95% CI, 1.01-2.29; *P* = .04) and nonresolving (aHR 2.30; 95% CI, 1.52-3.48; *P* < .001) AKI groups compared with hospitalized participants without AKI.

**Table 3.** Percentage of Participants Experiencing Renal Outcomes at 4 Years, by KDIGO Stage of AKI and AKI Recovery Patterns^a^

Outcome	No AKI	KDIGO Stage			AKI		*P* value^b^	
		1	2	3	Resolving	Nonresolving	Comparing the trend among KDIGO Stages	Comparing AKI recovery patterns
Death	12	22	22	24	22	22	.99	.73
CKD								
Incidence	11	28	36	34	24	39	.22	.002
Progression	9	24	33	45	22	31	.15	.10
Dialysis	2	6	8	4	6	7	.76	.58
MAKE	20	40	49	45	39	47	.14	.03

Abbreviations: AKI, acute kidney injury; CKD, chronic kidney disease; KDIGO, Kidney Disease: Improving Global Outcomes; MAKE, major adverse kidney events.

^a^ Kaplan-Meier estimates of the proportion of participants experiencing the renal outcomes at 4 years.

^b^ Comparing resolving with nonresolving AKI recovery subgroups or the trend among KDIGO stages of AKI.

**Table 4.** Association of AKI Recovery Patterns With MAKE^a^

AKI Subgroup	No. at risk	Events, No. (%)	Unadjusted		Model 1^b^		Model 2^c^	
			HR (95% CI)	*P* value	HR (95% CI)	*P* value	HR (95% CI)	*P* value
No AKI	769	192 (25)	1 [Reference]	NA	1 [Reference]	NA	1 [Reference]	NA
Resolving AKI	475	198 (42)	2.05 (1.68-2.50)	<.001	1.95 (1.58-2.40)	<.001	1.52 (1.01-2.29)	.04
Nonresolving AKI	294	160 (54)	2.90 (2.37-3.54)	<.001	2.80 (2.26-3.46)	<.001	2.30 (1.52-3.48)	<.001
Nonresolving AKI compared with resolving AKI	NA	NA	1.42 (1.16-1.78)	.001	1.44 (1.16-1.78)	<.001	1.51 (1.22-1.88)	<.001

Abbreviations: AKI, acute kidney injury; CKD, chronic kidney disease; HR, hazard ratio; MAKE, major adverse kidney events; NA, not applicable.

^a^ Composite of CKD incidence, CKD progression, dialysis, or death.

^b^ Adjusted for age, sex, black race, types 1 and 2 diabetes, CKD status, cardiovascular disease, sepsis, and site of study enrollment.

^c^ Additionally adjusted for Kidney Disease: Improving Global Outcomes stage of AKI at 72 hours after AKI diagnosis, shock, mechanical ventilation, and major surgery.

Within the AKI population, nonresolving AKI was associated with a 51% greater risk of MAKE (95% CI, 22%-88%; *P* < .001) compared with resolving AKI (Figure; eFigure in the Supplement). The higher risk of MAKE among patients with nonresolving AKI was due to a higher risk of incident CKD (aHR, 2.40; 95% CI, 1.65-3.49; *P* < .001) and progressive CKD (aHR, 1.58; 95% CI, 0.94-2.64; *P* = .07) compared with patients with resolving AKI (Figure; eTable 1 and eTable 2 in the Supplement). The risk of incident dialysis and death among patients was not significantly different between the AKI recovery patterns (eTable 3 and eTable 4 in the Supplement). There was no significant interaction between preadmission CKD status and AKI recovery trajectories for the risk of MAKE; AKI recovery subgroups were equally associated with MAKE among participants with CKD and participants without CKD (eTable 5 in the Supplement).

**Figure.**
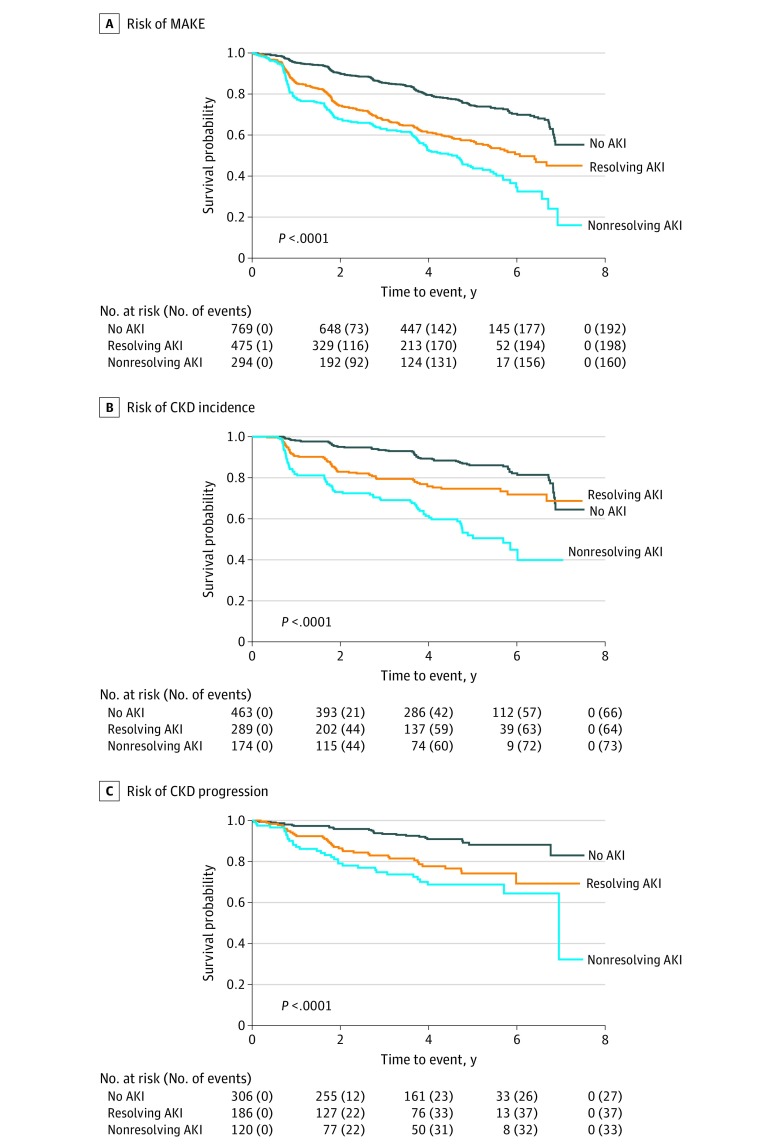
Risk of Renal Outcomes A, Kaplan-Meier plot demonstrates the highest risk for the composite outcome of major adverse kidney events (MAKE) among participants in the group with nonresolving acute kidney injury (AKI), with a stepwise decrease in the risk for MAKE in the group with resolving AKI, and then in participants without AKI. Major adverse kidney events are defined as the composite of chronic kidney disease (CKD) incidence, chronic kidney disease progression, initiation of long-term dialysis, or death from any cause during study follow-up. B, Risk of CKD incidence among patients without CKD at baseline. C, Risk of CKD progression among patients with CKD at baseline. The *P* value is a log-rank test of the null hypothesis that the survival distribution is the same across the no AKI, resolving AKI, and nonresolving AKI subgroups vs a significant difference in survival.

In sensitivity analyses, we found that the association between AKI recovery patterns and long-term outcomes was independent of length of hospital stay, initiation of vasopressors, and SCr concentrations at hospital discharge (eTables 6-8 in the Supplement). In a final sensitivity analysis, to account for violation of the Cox proportional hazards regression assumption, we determined the risk of MAKE stratified by 3 years of follow-up time. The risk of MAKE was consistently higher among participants with nonresolving AKI before or after 3 years of follow-up time compared with participants with no AKI or resolving AKI (eTable 9 in the Supplement).

## Discussion

A substantial body of literature demonstrates that AKI in the hospital setting is associated with risks for early mortality, and a growing body of literature suggests that patients with AKI also experience adverse kidney-specific long-term outcomes.^4,26,27^ However, AKI is common in hospitalized patients; therefore, clinicians are faced with prioritizing resources to closely monitor patients at high risk of CKD incidence or progression. Our report of a large, multiethnic cohort of patients hospitalized for at least 90 days for AKI shows a higher risk for MAKE among participants with resolving or nonresolving AKI compared with hospitalized participants without AKI. Moreover, there was a graded association, with the highest risk for MAKE in the group with nonresolving AKI, followed by the group with resolving AKI, and the least risk for MAKE among participants without AKI. Furthermore, when we controlled for the magnitude of increased SCr concentration (eg, KDIGO AKI severity staging or SCr concentrations at hospital discharge), the AKI recovery subgroups were still independently associated with long-term risk of MAKE. Finally, the baseline outpatient SCr concentraton prior to hospitalization and the maximum SCr concentration 72 hours after AKI diagnosis were not significantly different between the resolving and nonresolving AKI groups. Thus, the maximum increase in SCr concentration may be less important than the pattern of recovery in risk-stratified patients with AKI for future adverse kidney outcomes.

The use of AKI recovery subgroups to risk stratify patients with AKI we believe is clinically intuitive. The trajectory of SCr concentration incorporates serial measurements to overcome challenges in interpreting transient increases in SCr concentration that may be due to fluid resuscitation,^28^ medications,^29^ or brief kidney insults. Moreover, the increase in SCr concentration is dependent on the production of creatinine from skeletal muscle. Thus, an elderly person with minimal muscle mass but substantial kidney injury may not generate a large increase in SCr concentration but may still have a nonresolving recovery pattern. In contrast, a young, muscular patient with AKI from acute volume depletion may generate a large increase in SCr concentration with a resolving recovery pattern and, in turn, have a minimal increase in long-term complications. Thus, our results support the central hypothesis that the early recovery of the injured kidney is associated with improved kidney-specific clinical outcomes.

The implications of this work are 2-fold. First, our findings appear to be generalizable to most hospitalized patients who develop AKI. In epidemiologic studies, most patients who develop AKI will have KDIGO stage 1 AKI, and in-hospital mortality is uncommon (the mortality rate is ≤10%).^30,31,32,33^ Thus, 74% of patients in ASSESS-AKI had KDIGO stage 1 AKI, and patients were included if they survived 90 days after AKI diagnosis. We have shown that even patients with presumed mild AKI are at high risk for poor long-term kidney outcomes. However, current practice is for only a minority of patients with AKI to receive specialized nephrology follow-up at hospital discharge.^34^ One reason for the lack of follow-up may be the high rates of AKI in the hospital setting and the difficulty in identifying patients at highest risk for poor renal-specific outcomes. Previous studies have shown that SCr concentration at hospital discharge is associated with the development of CKD.^35^ We have now shown that prior to hospital discharge, the trajectory of SCr concentration within 72 hours after AKI diagnosis is associated with the development and progression of CKD independent of the SCr concentration at hospital discharge. Thus, AKI recovery subgroups could inform inpatient and outpatient nephrology consultation. Second, to our knowledge, no safe and effective pharmacotherapeutic strategies exist for treating AKI. One reason for the lack of therapies may be that grouping together patients with AKI with different risks for poor long-term outcomes may obscure a treatment signal specific to certain populations of patients with AKI.^10,36^ Identifying patients with nonresolving AKI may allow for prognostic enrichment of future AKI clinical trials (ie, selecting patients with a greater likelihood of the clinical event that the trial therapy is seeking to prevent).

### Strengths and Limitations

Our study has several strengths. First, ASSESS-AKI, a prospective study with predetermined end points, included a large, multiethnic population of hospitalized patients with long-term follow-up. Second, all patients included in ASSESS-AKI had a baseline SCr measurement prior to hospitalization. Patients who present to the hospital with community-acquired AKI may be underrecognized without a baseline SCr measurement. The benefit of prehospitalization SCr values appears to have allowed for the accurate classification of hospitalized patients with or without AKI. Third, all patients survived to at least 90 days after the index hospitalization. This study requirement addressed issues of competing risk because AKI has been shown to be associated with risk of hospital mortality.^19^ Fourth, physicians adjudicated clinical outcomes using rigorous consensus definitions. Fifth, our study outcome was the composite of kidney-specific adverse events over the study follow-up. The composite MAKE outcome incorporates several clinically important and patient-centered outcomes after AKI diagnosis.

The limitations of this study should be considered. First, given that this study enrolled patients surviving for at least 90 days after hospitalization, the results may not be generalizable to a population at high risk of inpatient death. However, the focus of this analysis was to assess the long-term implications of different AKI recovery patterns in hospitalized patients. Second, the trajectory of the SCr concentration may be a surrogate marker for length of stay or severity of illness. Although we attempted to correct for this possibility by adjusting for demographic characteristics, AKI risk factors, and KDIGO stage of AKI, residual confounding may still exist. Third, we had a relatively small number of eligible patients with KDIGO stage 2 or 3 AKI, so our results may not be fully generalizable to all such patients.

## Conclusions

We defined 2 AKI recovery subgroups (resolving and nonresolving) that exhibited differences regarding risk for long-term kidney-specific outcomes after hospitalization. In the future, AKI recovery subgroups may allow for improved risk stratification, facilitate prognostic enrichment of AKI clinical trials, and assist in targeting resources for follow-up and early detection of CKD in high-risk populations with AKI.

## Notes

Supplement. **eMethods.** Inclusion and Exclusion Criteria **eTable 1.** Association of AKI Recovery Subgroups With Development of CKD (Among Those Without CKD at Baseline) **eTable 2.** Association of AKI Recovery Subgroups With Progression of CKD (Among Those With CKD at Baseline) **eTable 3.** Association of AKI Recovery Subgroups With Risk of Death **eTable 4.** Association of AKI Recovery Subgroups With End-Stage Renal Disease (ESRD) **eTable 5.** Association of AKI Recovery Subgroups With MAKE (Composite of CKD Incidence, Chronic Dialysis or Death) Among Those Without CKD at Baseline **eTable 6.** Association of AKI Recovery Patterns With MAKE Including Hospital Length of Stay as a Covariate (Composite of CKD Incidence, CKD Progression, Dialysis or Death) **eTable 7.** Association of AKI Recovery Subgroups With MAKE Adjusting for Discharge SCr or KDIGO Stage of AKI at 72 Hours **eTable 8.** Association of AKI Recovery Patterns With MAKE Including Vasopressors as a Covariate Instead of Shock (Composite of CKD Incidence, CKD Progression, Dialysis or Death) **eTable 9.** Time-Stratified Association of AKI Subgroups With Composite Outcome (CKD Incidence, CKD Progression, Dialysis or Death) **eFigure.** Risk of MAKE Between Patients Without AKI, Resolving AKI and Nonresolving AKI With 95% CIs
