# Supplementary material for: Association Between Early Recovery of Kidney Function After Acute Kidney Injury and Long-term Clinical Outcomes
Source: JAMA Netw Open. 2020 Apr 13;3(4):e202682. doi: 10.1001/jamanetworkopen.2020.2682 (PMC7154800; doi:10.1001/jamanetworkopen.2020.2682)
Supplement: Supplement. — eMethods. Inclusion and Exclusion Criteria eTable 1. Association of AKI Recovery Subgroups With Development of CKD (Among Those Without CKD at Baseline) eTable 2. Association of AKI Recovery Subgroups With Progression of CKD (Among Those With CKD at Baseline) eTable 3. Association of AKI Recovery Subgroups With Risk of Death eTable 4. Association of AKI Recovery Subgroups With End-Stage Renal Disease (ESRD) eTable 5. Association of AKI Recovery Subgroups With MAKE (Composite of CKD Incidence, Chronic Dialysis or Death) Among Those Without CKD at Baseline eTable 6. Association of AKI Recovery Patterns With MAKE Including Hospital Length of Stay as a Covariate (Composite of CKD Incidence, CKD Progression, Dialysis or Death) eTable 7. Association of AKI Recovery Subgroups With MAKE Adjusting for Discharge SCr or KDIGO Stage of AKI at 72 Hours eTable 8. Association of AKI Recovery Patterns With MAKE Including Vasopressors as a Covariate Instead of Shock (Composite of CKD Incidence, CKD Progression, Dialysis or Death) eTable 9. Time-Stratified Association of AKI Subgroups With Composite Outcome (CKD Incidence, CKD Progression, Dialysis or Death) eFigure. Risk of MAKE Between Patients Without AKI, Resolving AKI and Nonresolving AKI With 95% CIs [file jamanetwopen-3-e202682-s001.pdf]

Supplementary Online Content

Bhatraju PK, Zelnick LR, Chinchilli VM, et al. Association between early recovery of kidney function after acute kidney injury and long-term clinical outcomes. *JAMA Netw Open.* 2020;3(4):e202682. doi:10.1001/jamanetworkopen.2020.2682

eMethods. Inclusion and Exclusion Criteria
eTable 1. Association of AKI Recovery Subgroups With Development of CKD (Among Those Without CKD at Baseline)
eTable 2. Association of AKI Recovery Subgroups With Progression of CKD (Among Those With CKD at Baseline)
eTable 3. Association of AKI Recovery Subgroups With Risk of Death
eTable 4. Association of AKI Recovery Subgroups With End-Stage Renal Disease (ESRD)
eTable 5. Association of AKI Recovery Subgroups With MAKE (Composite of CKD Incidence, Chronic Dialysis or Death) Among Those Without CKD at Baseline
eTable 6. Association of AKI Recovery Patterns With MAKE Including Hospital Length of Stay as a Covariate (Composite of CKD Incidence, CKD Progression, Dialysis or Death)
eTable 7. Association of AKI Recovery Subgroups With MAKE Adjusting for Discharge SCr or KDIGO Stage of AKI at 72 Hours
eTable 8. Association of AKI Recovery Patterns With MAKE Including Vasopressors as a Covariate Instead of Shock (Composite of CKD Incidence, CKD Progression, Dialysis or Death)
eTable 9. Time-Stratified Association of AKI Subgroups With Composite Outcome (CKD Incidence, CKD Progression, Dialysis or Death)
eFigure. Risk of MAKE Between Patients Without AKI, Resolving AKI and Nonresolving AKI With 95% CIs

This supplementary material has been provided by the authors to give readers additional information about their work.

© 2020 Bhatraju PK et al. *JAMA Network Open.*

eMethods. Inclusion and Exclusion Criteria

In ASSESS-AKI participants with and without AKI were initially matched on site and pre-admission chronic kidney disease (CKD) status, with additional matching using an integrated priority score based on age, prior cardiovascular disease or diabetes mellitus, pre-admission level of estimated glomerular filtration rate (eGFR) using the CKD-EPI equation, and ICU treatment during the index hospitalization.

Inclusion and exclusion criteria are listed below

**Inclusion Criteria**	**Exclusion Criteria**
Adult participants aged 18 years to 89 years	Inability to provide informed or surrogate consent
Documented "baseline" serum creatinine defined as the outpatient, non-emergency department test value nearest to the index hospitalization within 7 and 365 days prior to admission using an IDMS-traceable serum creatinine assay.	Died prior to the three-month study visit
At Yale, pre-operative serum creatinine results from an IDMS-traceable assay obtained within seven days before cardiac surgery can be used to define "baseline" kidney function for the subset of participants who are undergoing non-urgent cardiac surgery.	Enrolled in an active interventional study at the three-month in-person study visit.
	Actively pregnant or breastfeeding. Prior chronic hemodialysis, peritoneal dialysis (lasting ≥three months), or estimated GFR <15 ml/min/1.73 m2 not receiving renal replacement therapy. History of solid organ and/or hematopoietic cell transplants.

© 2020 Bhatraju PK et al. *JAMA Network Open.*

	Acute glomerulonephritis diagnosed clinically or by biopsy.
	Clinically significant urinary tract obstruction, confirmed by imaging.
	Hospitalization involving acute nephrectomy.
	History of multiple myeloma.
	Hepatorenal syndrome
	Metastatic cancer or systemic cancer receiving active treatment.
	New York Heart Association Class IV heart failure prior to index admission.
	Predicted survival of 12 months or less as determined by the participant's treating physician or Clinical Research.
	Center Principal Investigator.
	AKI participants who remain hospitalized 90 or more days after the AKI episode.
	ESRD at the time of the three-month study visit.
	Unable to provide research blood and urine samples

© 2020 Bhatraju PK et al. *JAMA Network Open.*

eTable 1. Association of AKI Recovery Subgroups With Development of CKD (Among Those Without CKD at Baseline)

			Unadjusted		Model 1		Model 2	
	N at risk	N events	HR (95% CI)	*P* value	HR (95% CI)	*P* value	HR (95% CI)	*P* value
No AKI	463	66 (14%)	1.00 (Ref.)		1.00 (Ref.)		1.00 (Ref.)	
Resolving AKI	289	64 (22%)	1.95 (1.38, 2.75)	< 0.001	2.08 (1.44, 2.98)	< 0.001	2.29 (1.20, 4.34)	0.01
Non-resolving AKI	174	73 (42%)	4.16 (3.04, 5.69)	< 0.001	4.21 (3.02, 5.86)	< 0.001	5.48 (2.97, 10.12)	< 0.001
Non-resolving AKI (compared with resolving AKI)			2.14 (1.51, 3.02)	< 0.001	2.03 (1.41, 2.91)	< 0.001	2.40 (1.65, 3.49)	< 0.001

Model 1: adjusted for age, sex, black race, diabetes, CKD, CVD, sepsis, and center. Model 2: additionally adjusted for maximum KDIGO stage of AKI at 72 hours after AKI diagnosis, shock, mechanical ventilation, major surgery. HR, hazard ratio; Ref, reference.

© 2020 Bhatraju PK et al. *JAMA Network Open.*

eTable 2. Association of AKI Recovery Subgroups With Progression of CKD (Among Those With CKD at Baseline)

			Unadjusted		Model 1		Model 2	
	**N at risk**	**N events**	**HR (95% CI)**	***P* value**	**HR (95% CI)**	***P* value**	**HR (95% CI)**	***P* value**
No AKI	306	27 (8.8%)	1.00 (Ref.)		1.00 (Ref.)		1.00 (Ref.)	
Resolving AKI	186	37 (20%)	2.68 (1.65, 4.35)	< 0.001	1.74 (1.02, 2.94)	0.04	2.08 (0.69, 6.33)	0.20
Non-resolving AKI	120	33 (28%)	3.84 (2.38, 6.22)	< 0.001	2.91 (1.75, 4.86)	< 0.001	3.28 (1.14, 9.42)	0.03
Non-resolving AKI (compared with resolving AKI)			1.43 (0.90, 2.29)	0.13	1.68 (1.02, 2.76)	0.04	1.58 (0.94, 2.64)	0.10

Model 1: adjusted for age, sex, black race, diabetes, CKD, CVD, sepsis, and center. Model 2: additionally adjusted for maximum KDIGO stage of AKI at 72 hours after AKI diagnosis, shock, mechanical ventilation, major surgery. HR, hazard ratio; Ref, reference.

© 2020 Bhatraju PK et al. *JAMA Network Open.*

eTable 3. Association of AKI Recovery Subgroups With Risk of Death

			Unadjusted		Model 1		Model 2	
	N at risk	N events	HR (95% CI)	*P* value	HR (95% CI)	*P* value	HR (95% CI)	*P* value
No AKI	769	112 (14%)	1.00 (Ref.)		1.00 (Ref.)		1.00 (Ref.)	
Resolving AKI	475	126 (27%)	2.03 (1.57, 2.61)	< 0.0001	1.91 (1.46, 2.50)	< 0.0001	1.60 (0.94, 2.71)	0.08
Non-resolving AKI	294	82 (28%)	2.08 (1.59, 2.72)	< 0.0001	2.03 (1.54, 2.69)	< 0.0001	1.76 (1.04, 2.98)	0.03
Non-resolving AKI (compared with resolving AKI)			1.03 (0.78, 1.35)	0.85	1.06 (0.80, 1.40)	0.67	1.11 (0.83, 1.47)	0.49

Model 1: adjusted for age, sex, black race, diabetes, CKD, CVD, sepsis, and center. Model 2: additionally adjusted for maximum KDIGO stage of AKI at 72 hours after AKI diagnosis, shock, mechanical ventilation, major surgery. HR, hazard ratio; Ref, reference.

© 2020 Bhatraju PK et al. *JAMA Network Open.*

eTable 4. Association of AKI Recovery Subgroups With End-Stage Renal Disease (ESRD)

			Unadjusted		Model 1		Model 2	
	N at risk	N events	HR (95% CI)	*P* value	HR (95% CI)	*P* value	HR (95% CI)	*P* value
No AKI	769	12 (1.6%)	1.00 (Ref.)		1.00 (Ref.)		1.00 (Ref.)	
Resolving AKI	475	28 (5.9%)	4.19 (2.24, 7.84)	< 0.001	2.53 (1.31, 4.89)	0.006	1.43 (0.37, 5.60)	0.61
Non-resolving AKI	294	18 (6.1%)	4.26 (2.05, 8.85)	< 0.001	3.15 (1.48, 6.73)	0.003	1.90 (0.51, 7.05)	0.34
Non-resolving AKI (compared with resolving AKI)			1.02 (0.56, 1.84)	0.96	1.25 (0.67, 2.33)	0.49	1.33 (0.70, 2.53)	0.39

Model 1: adjusted for age, sex, black race, diabetes, CKD, CVD, sepsis, and center. Model 2: additionally adjusted for maximum KDIGO stage of AKI at 72 hours after AKI diagnosis, shock, mechanical ventilation, major surgery. HR, hazard ratio; Ref, reference.

© 2020 Bhatraju PK et al. *JAMA Network Open.*

eTable 5. Association of AKI Recovery Subgroups With MAKE (Composite of CKD Incidence, Chronic Dialysis or Death) Among Those Without CKD at Baseline

			Unadjusted		Model 1		Model 2	
	**N at risk**	**N events**	**HR (95% CI)**	***P* value**	**HR (95% CI)**	***P* value**	**HR (95% CI)**	***P* value**
No AKI	463	66 (14%)	1.00 (Ref.)		1.00 (Ref.)		1.00 (Ref.)	
Resolving AKI	289	64 (22%)	1.91 (1.45, 2.50)	< 0.001	1.88 (1.41, 2.50)	< 0.001	1.63 (0.95, 2.80)	0.07
Non-resolving AKI	174	73 (42%)	2.99 (2.29, 3.90)	< 0.001	3.01 (2.27, 3.99)	< 0.001	3.00 (1.77, 5.10)	< 0.001
Non-resolving AKI compared with resolving AKI			1.57 (1.18, 2.09)	0.002	1.60 (1.19, 2.15)	0.002	1.84 (1.36, 2.50)	< 0.001

Model 1: Adjusted for age, sex, black race, diabetes, CKD, CVD, sepsis, and center. Model 2: additionally adjusted for maximum KDIGO stage of AKI at 72 hours post AKI diagnosis, shock, mechanical ventilation, major surgery. HR, hazard ratio; Ref, reference.

© 2020 Bhatraju PK et al. *JAMA Network Open.*

eTable 6. Association of AKI Recovery Patterns With MAKE Including Hospital Length of Stay as a Covariate (Composite of CKD Incidence, CKD Progression, Dialysis or Death)

			**Unadjusted**		**Model 1**		**Model 2**	
	**N at risk**	**N events**	**HR (95% CI)**	***P* value**	**HR (95% CI)**	***P* value**	**HR (95% CI)**	***P* value**
No AKI	769	192 (25%)	1.00 (Ref.)		1.00 (Ref.)		1.00 (Ref.)	
Resolving AKI	475	198 (42%)	2.05 (1.68, 2.50)	< 0.001	1.95 (1.58, 2.40)	< 0.001	1.47 (0.96, 2.25)	0.08
Non-resolving AKI	294	160 (54%)	2.90 (2.37, 3.54)	< 0.001	2.80 (2.26, 3.46)	< 0.001	2.17 (1.42, 3.31)	< 0.001
Non-resolving AKI compared with resolving AKI			1.42 (1.15, 1.75)	0.001	1.44 (1.16, 1.78)	0.001	1.48 (1.18, 1.85)	< 0.001

Model 1: Adjusted for age, sex, black race, diabetes, CVD, and sepsis. Model 2: additionally adjusted for maximum KDIGO stage of AKI at 72 hours post AKI diagnosis, shock, mechanical ventilation, major surgery, hospital length of stay. HR, hazard ratio; Ref, reference.

© 2020 Bhatraju PK et al. *JAMA Network Open.*

eTable 7. Association of AKI Recovery Subgroups With MAKE Adjusting for Discharge SCr or KDIGO Stage of AKI at 72 Hours

	**Model 1a (adjusting for hospital discharge serum creatinine concentration)**		**Model 1b (KDIGO stage of AKI at 72 hours)**	
	**HR (95% CI)**	***P* value**	**HR (95% CI)**	***P* value**
No AKI	1.00 (Ref.)		1.00 (Ref.)	
Resolving AKI	1.80 (1.47, 2.22)	< 0.001	1.55 (1.03, 2.33)	0.04
Non-resolving AKI	2.37 (1.87, 3.00)	< 0.001	2.37 (1.57, 3.58)	< 0.001
Non-resolving AKI compared with resolving AKI	1.31 (1.05, 1.64)	0.02	1.53 (1.23, 1.90)	< 0.001

Model 1a: Adjusted for age, sex, black race, diabetes, CKD status, CVD, sepsis, center, shock, mechanical ventilation, major surgery and serum creatinine concentration at hospital discharge.

Model 1b: Adjusted for age, sex, black race, diabetes, CKD status, CVD, sepsis, center, shock, mechanical ventilation, major surgery and maximum KDIGO stage of AKI at 72 hours post AKI diagnosis.

HR, hazard ratio; Ref, reference.

© 2020 Bhatraju PK et al. *JAMA Network Open.*

eTable 8. Association of AKI Recovery Patterns With MAKE Including Vasopressors as a Covariate Instead of Shock (Composite of CKD Incidence, CKD Progression, Dialysis or Death)

			**Unadjusted**		**Model 1**		**Model 2**	
	**N at risk**	**N events**	**HR (95% CI)**	***P* value**	**HR (95% CI)**	***P* value**	**HR (95% CI)**	***P* value**
No AKI	769	192 (25%)	1.00 (Ref.)		1.00 (Ref.)		1.00 (Ref.)	
Resolving AKI	475	198 (42%)	2.05 (1.68, 2.50)	< 0.001	1.95 (1.58, 2.40)	< 0.001	1.54 (1.03, 2.30)	0.04
Non-resolving AKI	294	160 (54%)	2.90 (2.37, 3.54)	< 0.001	2.80 (2.26, 3.46)	< 0.001	2.32 (1.54, 3.50)	< 0.001
Non-resolving AKI compared with resolving AKI			1.42 (1.15, 1.75)	0.001	1.44 (1.16, 1.78)	0.001	1.51 (1.21, 1.87)	< 0.001

Model 1: Adjusted for age, sex, black race, diabetes, CKD, CVD, sepsis, and center. Model 2: additionally adjusted for maximum KDIGO stage of AKI at 72 hours post AKI diagnosis, vasopressors, mechanical ventilation, major surgery. HR, hazard ratio; Ref, reference.

© 2020 Bhatraju PK et al. *JAMA Network Open.*

eTable 9. Time-Stratified Association of AKI Subgroups With Composite Outcome (CKD Incidence, CKD Progression, Dialysis or Death)

			Unadjusted		Model 1		Model 2	
	**N at risk**	**N events**	**HR (95% CI)**	***P* value**	**HR (95% CI)**	***P* value**	**HR (95% CI)**	***P* value**
**Before 3 years**								
No AKI	769	105	1.00 (Ref.)		1.00 (Ref.)		1.00 (Ref.)	
Resolving AKI	475	145	1.42 (1.10, 1.83)	0.007	1.46 (1.12, 1.89)	0.005	1.25 (0.80, 1.95)	0.33
Non-resolving AKI	294	105	2.11 (1.61, 2.77)	< 0.001	2.12 (1.60, 2.80)	< 0.001	1.81 (1.15, 2.85)	0.01
Non-resolving AKI compared with resolving AKI			1.49 (1.16, 1.92)	0.002	1.45 (1.12, 1.88)	0.005	1.45 (1.12, 1.89)	0.006
**After 3 years**								
No AKI	587	87	1.00 (Ref.)		1.00 (Ref.)		1.00 (Ref.)	
Resolving AKI	281	52	1.36 (0.97, 1.92)	0.08	1.32 (0.93, 1.87)	0.12	1.12 (0.68, 1.86)	0.65
Non-resolving AKI	171	55	2.72 (1.93, 3.83)	< 0.001	2.65 (1.88, 3.74)	< 0.001	2.19 (1.34, 3.60)	0.002

© 2020 Bhatraju PK et al. *JAMA Network Open.*

Non-resolving AKI compared with resolving AKI			2.00 (1.36, 2.92)	< 0.001	2.00 (1.37, 2.94)	< 0.001	1.95 (1.33, 2.87)	< 0.001

Model 1: Adjusted for age, sex, black race, diabetes, CKD status, CVD, sepsis, and center. Model 2: additionally adjusted for maximum KDIGO stage of AKI at 72 hours post AKI diagnosis, shock, mechanical ventilation, major surgery. HR, hazard ratio; Ref, reference.

© 2020 Bhatraju PK et al. *JAMA Network Open.*

eFigure. Risk of MAKE Between Patients Without AKI, Resolving AKI and Nonresolving AKI With 95% CIs

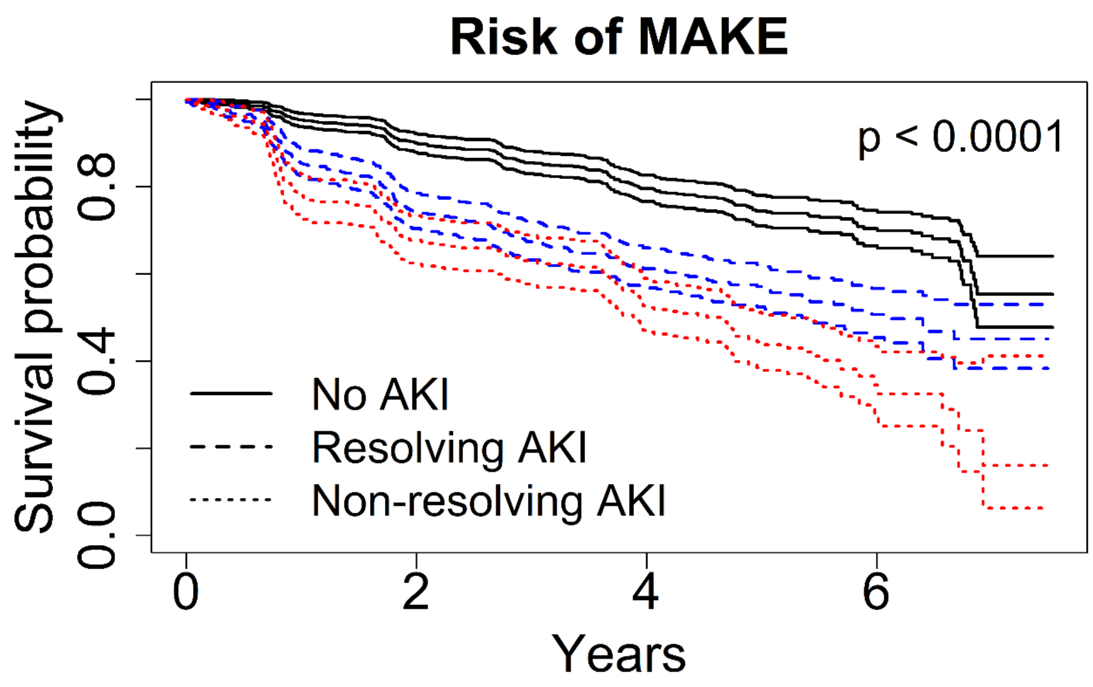

Number at risk (number of events)

No AKI	769 (0)	648 (73)	447 (142)	145 (177)	0 (192)
Resolving	475 (1)	329 (116)	213 (170)	52 (194)	0 (198)
Non-resolving	294 (0)	192 (92)	124 (131)	17 (156)	0 (160)

© 2020 Bhatraju PK et al. *JAMA Network Open.*
